# Diesel exhaust particles alter mitochondrial bioenergetics and cAMP producing capacity in human bronchial epithelial cells

**DOI:** 10.3389/ftox.2024.1412864

**Published:** 2024-07-25

**Authors:** Isabella Cattani-Cavalieri, Marina Trombetta-Lima, Hong Yan, Ana L. Manzano-Covarrubias, Hoeke A. Baarsma, Asmaa Oun, Melissa Mol van der Veen, Emily Oosterhout, Amalia M. Dolga, Rennolds S. Ostrom, Samuel Santos Valenca, Martina Schmidt

**Affiliations:** ^1^ Department of Molecular Pharmacology, University of Groningen, Groningen, Netherlands; ^2^ Groningen Research Institute for Asthma and COPD (GRIAC), University Medical Center Groningen, University of Groningen, Groningen, Netherlands; ^3^ Institute of Biomedical Sciences, Federal University of Rio de Janeiro, Rio de Janeiro, Brazil; ^4^ Department of Biomedical and Pharmaceutical Sciences, School of Pharmacy, Chapman University, Irvine, CA, United States; ^5^ Department of Pharmaceutical Technology and Biopharmacy, University of Groningen, Groningen, Netherlands

**Keywords:** lung, air pollution, diesel exhaust particles, mitochondria, oxidative stress, cAMP

## Abstract

**Introduction:** Air pollution from diesel combustion is linked in part to the generation of diesel exhaust particles (DEP). DEP exposure induces various processes, including inflammation and oxidative stress, which ultimately contribute to a decline in lung function. Cyclic AMP (cAMP) signaling is critical for lung homeostasis. The impact of DEP on cAMP signaling is largely unknown.

**Methods:** We exposed human bronchial epithelial (BEAS-2B) cells to DEP for 24–72 h and evaluated mitochondrial bioenergetics, markers of oxidative stress and inflammation and the components of cAMP signaling. Mitochondrial bioenergetics was measured at 72 h to capture the potential and accumulative effects of prolonged DEP exposure on mitochondrial function.

**Results:** DEP profoundly altered mitochondrial morphology and network integrity, reduced both basal and ATP-linked respiration as well as the glycolytic capacity of mitochondria. DEP exposure increased gene expression of oxidative stress and inflammation markers such as interleukin-8 and interleukin-6. DEP significantly affected mRNA levels of exchange protein directly activated by cAMP-1 and -2 (Epac1, Epac2), appeared to increase Epac1 protein, but left phospho-PKA levels unhanged. DEP exposure increased A-kinase anchoring protein 1, β_2_‐adrenoceptor and prostanoid E receptor subtype 4 mRNA levels. Interestingly, DEP decreased mRNA levels of adenylyl cyclase 9 and reduced cAMP levels stimulated by forskolin (AC activator), fenoterol (β_2_-AR agonist) or PGE2 (EPR agonist).

**Discussion:** Our findings suggest that DEP induces mitochondrial dysfunction, a process accompanied by oxidative stress and inflammation, and broadly dampens cAMP signaling. These epithelial responses may contribute to lung dysfunction induced by air pollution exposure.

## Introduction

Air pollution is a major environmental threat to human health and has been linked to various lung disorders such as asthma and COPD, particularly in patient groups suffering from exacerbations (Kunzli and Tager, 2005; Prüss-Üstün et al., 2016; Choi et al., 2018; Shin et al., 2020). The diesel engine is considered the most prevalent source of pollution and is widely used in automotive transportation (Steiner et al., 2016). The main pollutants emitted by diesel engines are carbon oxides (CO and CO_2_), sulfur oxides, nitrogen oxides and polycyclic aromatic hydrocarbons (Gioda et al., 2011). In addition to the release of gaseous pollutants, diesel combustion generates diesel exhaust particles (DEP). The main compounds found in DEP are metals and polycyclic aromatic hydrocarbons, both highly toxic to health (Wichmann, 2007; EPA and McCarthy, 2010).

Various environmental insults lead to mitochondrial dysfunction, a process known to accelerate cell damage and limit cell viability, in lung (Prakash et al., 2017). The mitochondrial electron transport chain (ETC) is composed of complexes I–IV, which have been linked to the production of mitochondrial ROS. Under physiological conditions, some electrons in the ETC leak out and interact with molecular oxygen, rendering relatively low levels of superoxide anion radicals (Turrens, 2003). Under pathophysiological conditions, such as cardiovascular disease, COPD, and cancer, there is excess generation of the superoxide anion (Zimmerman et al., 2004; Marin-Corral et al., 2009; Indo et al., 2015). Importantly, exposure of mice to DEP caused lung injury and substantially reduced mitochondrial function (Hiura et al., 2000; Zhao et al., 2009). Exposure to urban particulate matter not only heightened mitochondrial ROS levels but also significantly altered crucial mitochondrial functions within primary human olfactory mucosal cells (Chew et al., 2020). Particulate matter exposure induced mitochondrial ROS in cultured mouse lung epithelial cells leading to mitochondrial oxidative stress (Soberanes et al., 2012). Thus, mitochondrial dysfunction may play a central role in the induction of pulmonary impairments in response to DEP.

Several lines of evidence indicate that DEP promotes the induction of inflammatory mediators such as interleukin-6 (IL-6) and IL-8 (Zuo et al., 2019a; Cattani-Cavalieri et al., 2020). Exposure of human bronchial epithelial (BEAS-2B) cells to DEP (generated by an unloaded diesel engine) time-dependently upregulated CXCL8 and IL-6 mRNA expression (Bach et al., 2014). Moreover, exposure of rats to diesel engine exhaust increased IL-8 and IL-6 protein in serum and bronchoalveolar lavage fluid (Wang et al., 2019). In addition to inflammation, DEP induces oxidative stress characterized by an imbalance between the production of oxidants and antioxidant defense systems, subsequently leading to an overproduction of reactive oxygen species (ROS) (Nemmar et al., 2015; Farina et al., 2017). This process involves several transcription factors, including hypoxia inducible factor-1α (HIF-1α) and nuclear respiratory factor 1 (Nrf1) (Zhang et al., 2011; Mavrofrydi and Papazafiri, 2012; Zhao et al., 2014). Furthermore, thioredoxins, heme oxygenase-1 and -2 (HO-1, HO-2), glutathione peroxidase-1 (Gpx-1), catalase (CAT), and superoxide dismutase-1 and -2 (SOD-1, SOD-2) protect cells against oxidative stress based on their antioxidant properties (Lee et al., 2013; Ighodaro and Akinloye, 2018), and thereby maintain a balanced cellular redox potential. ROS production increases in Type II human alveolar epithelial cells exposed to diesel particulate matter (Patel et al., 2011). Moreover, exposure of RAW 264.7 macrophages to a methanol extract of DEP from a light-duty diesel source elevated superoxide and hydrogen peroxide, which was accompanied by the induction of apoptosis (Hiura et al., 2000). Exposure of BEAS-2B cells to ultrafine particles derived from diesel elevated mRNA expression of HO-1 and thioredoxin (Grilli et al., 2018).

Air pollutants may alter cAMP signaling, which is known to diminish inflammation and oxidative stress (Zuo et al., 2019a). The production of cAMP is induced by activation of Gs‐coupled receptors, such as the β_2_‐adrenoceptor (β_2_-AR) and prostanoid E receptors (EP) subtypes, and subsequent activation of adenylyl cyclases (ACs) (Poppinga et al., 2014). Intracellular cAMP levels are tightly controlled by phosphodiesterases (PDEs) (Zuo et al., 2019b). Profound alterations in the expression profiles of both PDEs and cAMP-stimulating receptors have been reported in various diseases (Insel et al., 2019; Jones et al., 2019; Zuo et al., 2020a; Haak et al., 2020). cAMP alters cell function via effectors such as protein kinase A (PKA) and exchange protein directly activated by cAMP (Epac). Furthermore, cAMP signaling is coordinated by different members of the A-kinase anchoring protein (AKAP) family; PKA and Epac can associate with various AKAPs, some of which tether these effectors to the mitochondria where they regulate mitochondrial function and cellular homeostasis (Poppinga et al., 2014; Poppinga et al., 2015). The AKAP family member AKAP1 acts as a mitochondrial scaffold protein involved in mitochondrial cAMP compartmentalization and is known to regulate mitochondrial function (Merrill and Strack, 2014).

Studies have investigated the influence of cAMP and its effectors on the regulation of mitochondrial function, metabolism, signaling and ROS production. The oxidative phosphorylation system (OXPHOS), responsible for energy release within mitochondria, is closely associated with ROS generation during mitochondrial activity. The augmentation of OXPHOS, located in the mitochondrial inner membrane, has been linked to cAMP/PKA signaling, indirectly suggesting a regulatory role of cAMP/PKA in mitochondrial ROS production (Papa et al., 1996; Acin-Perez et al., 2009). Moreover, elevated intracellular cAMP levels enhance mitochondrial enzyme activities and the abundance of mitochondrial cytochromes (Yoboue et al., 2012). Consequently, the ability of cAMP to modulate OXPHOS suggests its involvement in the maintenance of energy storage, consumption and ROS production within mitochondria. We reported previously that β_2_-ARs, EP receptors, Epac, and AKAPs are linked to inflammation and oxidative stress triggered by noxious particles including air pollutants (Oldenburger et al., 2014a; Poppinga et al., 2015).

Air pollution causes mitochondrial dysfunction, accompanied with airway inflammation and oxidative stress, processes which may trigger alterations in cAMP signaling. However, the exact effects and associated molecular mechanisms of DEP on mitochondrial biodynamics and cAMP-mediated signaling remain ill-defined. Here, we report that DEP exposure leads to profound alterations in mitochondrial function and a reduction of AC9 expression that correlates with decreased cAMP-producing capacity in human bronchial epithelial cells.

## Methods and materials

### Cell culture

Human bronchial epithelial cells (BEAS-2B cell line) from ATCC were maintained in a humidified incubator of 5% (v/v) CO_2_ at 37°C, using RPMI 1640 (Lonza) supplemented with 10% v/v heat-inactivated fetal bovine serum (FBS) (Biowest) and 1% of antibiotics (penicillin/streptomycin) (Gibco) (Zuo et al., 2020b). Cells were detached from the flask with trypsin/EDTA (Gibco) and seeded in appropriate cell culture plates. Cells were maintained in 1% v/v FBS medium and antibiotics (penicillin 100 U/mL, streptomycin 100 μg/mL) (Gibco) 24 h before and during exposure to DEP or other factors.

### Cell exposure to DEP

BEAS-2B cells were exposed to 100 or 300 μg/mL DEP (SRM 2975, National Institute of Standards and Technology- NIST) for 24, 48 or 72 h; concentrations and exposure times were based on literature on this topic (Bach et al., 2014; Vattanasit et al., 2014; Rynning et al., 2018). Prior to each set of experiments, a fresh stock solution of 1 mg/mL DEP was prepared using RPMI medium with 10% FBS, 1% of penicillin/streptomycin and 0.5% DMSO. Exposure to 0.5% DMSO served as control. The “Certificate of Analysis” provided by NIST can be found in the following link: https://www-s.nist.gov/srmors/certificates/2975.pdf.

### Cell viability assay and xCELLigence measurement

Cell viability was quantified by colorimetric 3-(4,5-Dimethylthiazol-2-yl)-2,5-Diphenyltetrazolium Bromide (MTT) reduction assay. MTT solution was added in a final concentration of 0.5 mg/mL to the cells in a 96-well plate with 10,000 cells/well for 1 h at 37°C. Next, the MTT-containing medium was removed and the plate was incubated at −20°C for at least 1 h. Following the incubation, the resulting formazan was dissolved in DMSO for 1 h at 37°C under continuous shaking conditions (Zuo et al., 2020b). The absorbance was measured at 570 versus 630 nm with a Synergy™H1 (Bad Friedrichshall, Germany) Hybrid Multi-Mode Reader.

The xCELLigence system was used to evaluate real-time cell viability for quantitative assessment (Zuo et al., 2020b). For these experiments, the cells were pretreated with DEP and reseeded. Cellular impedance was determined and normalized to the time of treatment represented as Normalized Cell Index (NCI), which was relative to the last value control for each experiment. NCI is defined as the starting point (t = 0 h) of the experiment.

### Real-time quantitative PCR

BEAS-2B cells were seeded in a 6-well plate with 400,000 cells/well. Total RNA was extracted from cells using TRIZOL reagent (TRI Reagent Solution, Applied Biosystems, the Netherlands) according to manufacturer’s protocol. The total RNA yield was determined by a NanoDrop 1,000 Spectrophotometer (Thermo Fisher Scientific, Wilmington, DE, United States ). Equal amounts of RNA were used to synthesize cDNA and RT-qPCR was carried out in the presence of SYBR Green using an Illumina Eco Real-Time PCR system. PCR cycling was performed by denaturation at 94°C for 30 s, annealing at 59°C for 30 s, and extension at 72°C for 30 s for 45 cycles (Zuo et al., 2020b). RT-qPCR data was analyzed with LinRegPCR software. To analyze RT-qPCR data, the amount of target gene was normalized to the reference genes 18 S ribosomal RNA, SDHA, and RPL13A. The Cq mean ± SD from control group is listed in Supplementary Table S1. Primer sequences are listed in Supplementary Table S2.

### Western blotting

BEAS-2B cells were seeded in a 6-well plate with 400,000 cells/well. Cellular protein was collected with RIPA buffer (65 mM Tris, 155 mM NaCl, 1% Igepal CA‐630, 0.25% sodium deoxycholate, 1 mM EDTA, pH 7.4 and a mixture of protease inhibitors: 1 mM Na3VO4, 1 mM NaF, 10 μg/mL leupetin, 10 μg/mL pepstatin A, 10 μg/mL aprotinin) and total protein content was quantified by BCA protein assay (Pierce). Equal amounts of total protein were separated by 10% SDS–polyacrylamide gel electrophoresis and subsequently transferred to nitrocellulose membranes. After blocking the membranes with Roti-Block (Carl Roth, Karlsruhe, Germany), Epac1 and 2 (1:1,000, Cell Signaling Technology), phospho-PKA (1:1,000, Cell Signaling Technology), HIF-1α (1:1,000, Cell Signaling Technology) and GAPDH (1:3,000, Sigma) were applied overnight at 4°C. After thorough washing, the membranes were incubated with secondary antibody (anti-mouse IgG, 1: 5,000; anti-rabbit IgG, 1: 5,000) at room temperature (RT) for 2 h (Zuo et al., 2020b). The antigen-antibody complexes were detected using a Western detection ECL-plus kit (PerkinElmer, Waltman, MA). ImageJ software was used for densitometric analysis of the bands; expression was normalized to GAPDH.

### Immunofluorescence

Immunofluorescence was performed on BEAS-2B cells. BEAS-2B cells were seeded, cultured and exposed as described above. The cells were fixed with 4% paraformaldehyde at −20°C. BEAS-2B were washed 3 times with PBS and then blocked with 1% (w/v) BSA/PBS. Primary antibodies for Epac1 and 2 (1:200, Cell Signaling) were applied overnight at 4°C. After washing, cultures were subjected to secondary antibody [Alexa Fluor 488 nm donkey anti-goat (1:2,000) and Cy™3 AffiniPure donkey anti-mouse (1:2,000), Jackson, Cambridgeshire, United Kingdom] for 2 h (Zuo et al., 2020b). Slides were mounted with mounting medium containing DAPI (Abcam, Cambridge, United Kingdom). Images were captured with a Leica DM4000b Fluorescence microscope (Leica Microsystems, Germany) equipped with a Leica DFC 345 FX camera (10× objective lens).

### Enzyme-linked immunosorbent assays (ELISA)

BEAS-2B cells were exposed to 100 or 300 μg/mL DEP (SRM 2975, National Institute of Standards and Technology) for 24 h. Culture medium was collected to measure IL-8 and IL-6 protein concentrations using specific enzyme-linked immunosorbent assays (ELISAs) (Zuo et al., 2020b). Data are presented as pg/mL.

### Morphological characterization and quantification of mitochondria

BEAS-2B cells were seeded on coverslips and exposed to DEP as described previously. After fixation at RT by using 4% paraformaldehyde, the cells were co-stained with 200 nM MitoTracker Deep Red (Invitrogen) for mitochondria and DAPI (4′,6-diamidino-2-phenylindole) (Abcam) for the nuclei. Following incubation with MitoTracker Deep Red, cells were washed with 1x PBS. For morphological characterization and quantification, mitochondria were manually grouped in 3 distinct categories. Category I consisted of elongated mitochondria that were distributed throughout the cell, category II contained fragmented mitochondria, and category III mitochondria exhibited an undecided morphology (Grohm et al., 2010; Dolga et al., 2013). Mitochondrial footprint, branching, and average branch length were determined by the semi-automated analysis of mitochondrial networks using the Mitochondrial Network Analysis (MiNA) macro for ImageJ (Schneider et al., 2012; Valente et al., 2017). For technical reasons the MitoTracker experiments were only performed in cells exposed to 100 μg/mL DEP for 24 h. Images were obtained by Nikon Inverted Research Fluorescence Microscope ECLIPSE Ti2-E (60× objective lens).

### Seahorse XF analysis

The oxygen consumption rate (OCR) and extracellular acidification rate (ECAR) were measured with the Seahorse system. BEAS-2B cells were seeded in Seahorse XF 96-well plates (Seahorse Biosystems, Agilent Technologies, Waldbronn, Germany) with 10,000 cells/well and exposed to 100 or 300 μg/mL DEP for 72 h. On the day of the measurement, medium was removed, and cells were incubated in assay medium supplemented with 4.5 g/L glucose, 2 mM glutamine, and 1 mM pyruvate (pH 7.35) at 37°C (without CO_2_) for 1 h. During the assay, basal respiration was measured before sequentially injecting the following inhibitors: oligomycin (2 µM), FCCP (carbonyl cyanide-4 (trifluoromethoxy) phenylhydrazone, 0.5 µM) and rotenone (1 µM)/antimycin A (1 µM). Oligomycin is an ATP synthase inhibitor that changes the cellular energy production to glycolysis, FCCP is an uncoupling agent of oxygen consumption which stimulates the OCR, and the rotenone/antimycin A combination inhibits complex I/III. After injection of each compound, OCR and ECAR were determined (Sabogal-Guaqueta et al., 2019). ATP rate index (MitoATP/GlycoATP) and ATP production rate were determined based on the ECAR profile. The OCR and ECAR measurements were normalized to the protein amount in each well (determined by BCA assay).

### Real-time cAMP measurements

For real-time cAMP measurements, BEAS-2B cells were seeded on 96-well plates with 10.000 cells/well according to manufacturer’s protocol. After culturing, cells were transiently transfected with the GloSensor cAMP plasmid (2 μg), which was added with RNAiMAX Transfection Reagent. The cells were maintained in a humidified incubator (5% (v/v) CO_2_, 37°C) for 24 h. The GloSensor assay was carried out as per manufacturer’s instructions (Promega, Madison, WI, United States ). In brief, cells were incubated in RPMI 1640 L-glutamine and 25 mM HEPES supplemented with 10% FBS v/v containing 2% GloSensor cAMP reagent for 2 h at RT (Rosethorne et al., 2010). Luminescence was measured and continuously monitored over time, with 1 read per well every 3 min, following the addition of forskolin (10 µM), fenoterol (10 µM) or the PGE2 analogue 16,16-dimethyl prostaglandin E2 (3 µM).

### Statistical analysis

All data are presented as mean ± SD. At least 3 independent experiments were conducted for each treatment. Using Graph Pad Prism software (Graph Pad Software, San Diego, CA, United States ), the data were compared using Student’s t-test for statistical comparisons between two groups or ANOVA for multiple comparisons followed by Newman-Keuls comparison test. In all instances, *p* < 0.05 was considered statistically significant.

## Results

### DEP 100 μg/mL affects mitochondrial morphology

**FIGURE 1:**
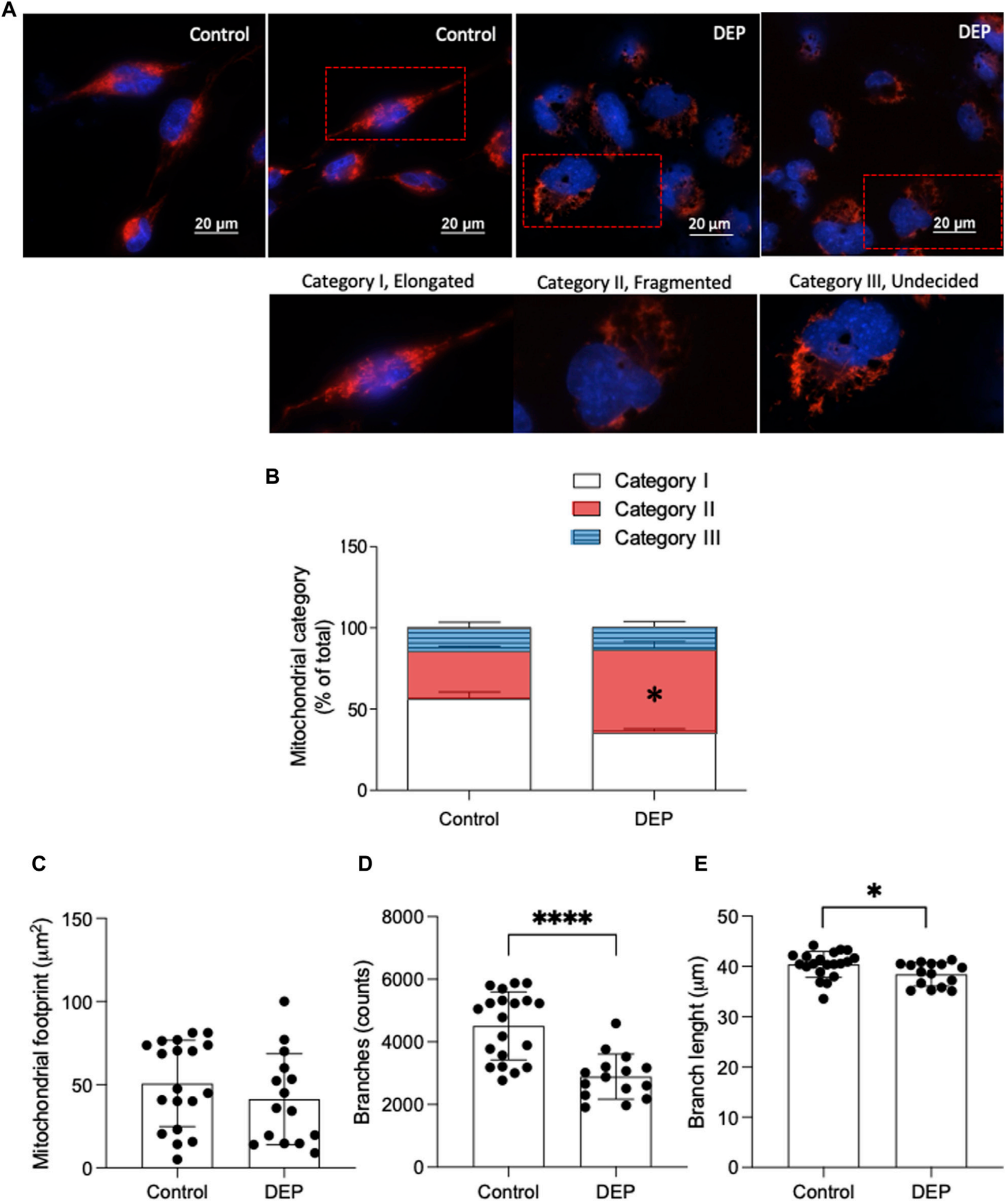
Exposure of BEAS-2B cells to DEP alters mitochondrial morphology. **(A)** Representative MitoTracker images are shown. Scale bar of 20 μm. **(B)** Relative quantification of mitochondria in categories I-III. **(C)** Mitochondrial footprint (µm^2^), **(D)** branches (counts) and **(E)** branch length was analyzed using the ImageJ MINA-Macro. BEAS-2B cells were exposed to 100 μg/mL DEP or 0.5% DMSO (control). Data represent 4 independent experiments (15–20 technical replicates per group) and are expressed as mean ± SD; *p < 0.05 and ****p < 0.0001 significant difference between indicated groups.

Mitochondrial dysfunction is recognized as a fundamental pathological mechanism in several chronic diseases, including COPD and asthma (Cloonan and Choi, 2016). We first explored the impact of DEP exposure on mitochondrial morphology to understand if this type of air pollution could alter mitochondrial function. Using MitoTracker Deep Red, we investigated whether exposure to DEP altered mitochondrial morphology (Dolga et al., 2013). Mitochondria were grouped in one of three categories: I, elongated mitochondria; II, fragmented mitochondria; and III, mitochondria with an unclear morphology. We observed that exposure to 100 μg/mL DEP for 24 h promoted a switch from category I to II, while relative numbers in category III remained the same (Figures 1A, B). Using the ImageJ MINA macro, we investigated whether exposure to DEP altered the mitochondrial network integrity. DEP exposure for 24 h did not significantly decrease the mitochondrial footprint. However, the number of branches and branch length was reduced by DEP exposure for 24 h (Figures, 1C–E). Since mitochondrial morphology is directly correlated to mitochondrial function (Mannella, 2006; Perkins and Ellisman, 2011; Picard et al., 2013; Siegmund et al., 2018), these data suggest that exposure of BEAS-2B cells to DEP interferes with mitochondrial function.

### DEP alters mitochondrial respiration and glycolysis

**FIGURE 2:**
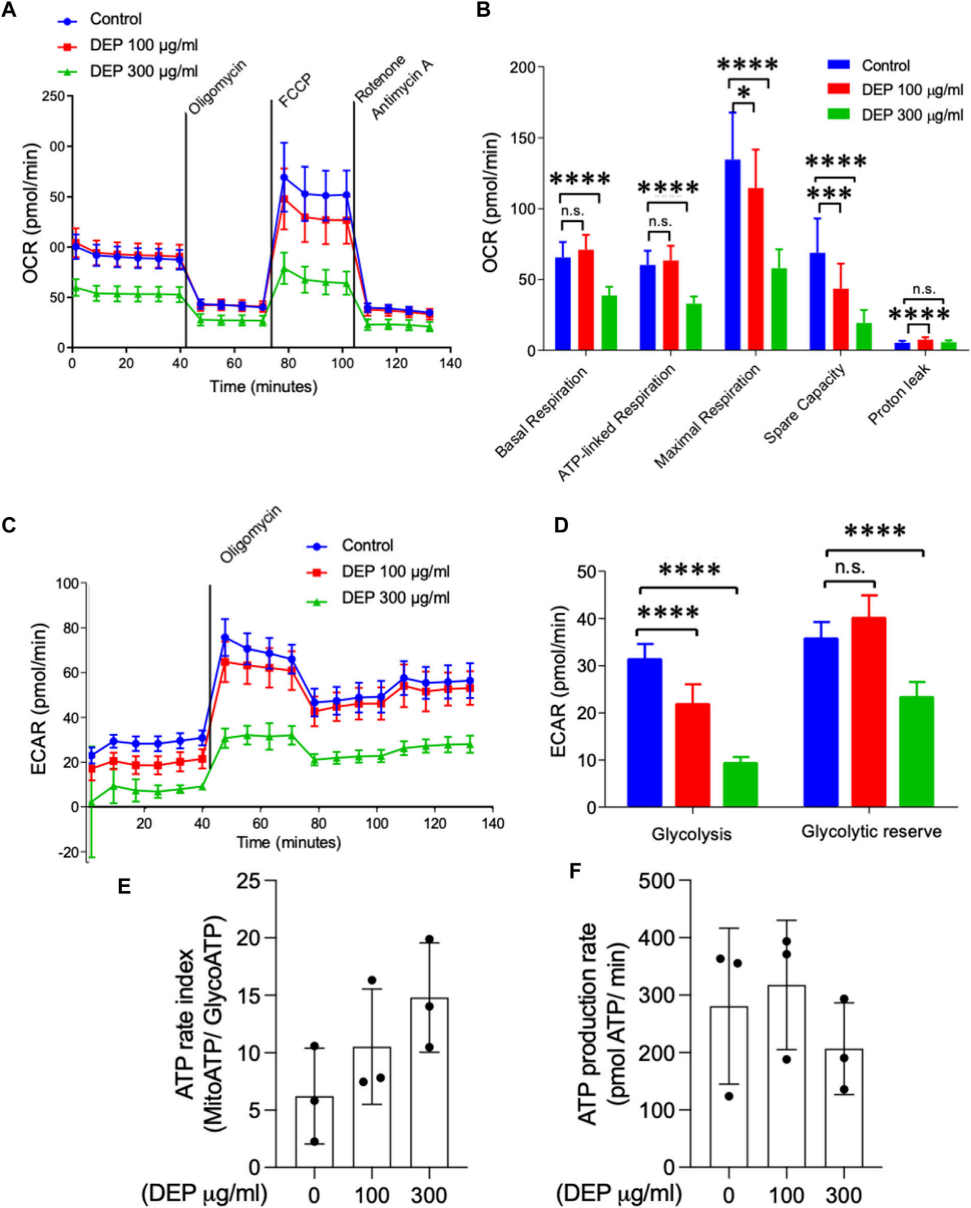
Exposure of BEAS-2B cells to DEP negatively affects mitochondrial respiration and glycolysis. The injection of oligomycin was used to inhibit ATP synthase (complex V), leading to a reduction in mitochondrial respiration. The parameters measured after oligomycin injection are indicated as ATP-linked respiration (ATP produced by the mitochondria) and proton leak (remining basal respiration not coupled to ATP production). FCCP was then used as an uncoupling agent, which stimulates the respiratory chain to operate at maximum capacity, revealing the maximum rate of respiration of the cell. Spare capacity shows the ability of the cell to respond to an energetic demand. Rotenone and actinomycin were then used to inhibit complex I and III, respectively, shutting down mitochondrial respiration (Divakaruni et al., 2014) **(A,B)** Basal respiration, ATP-linked respiration, maximal respiration and spare capacity were analyzed using Seahorse analyzer. **(C)** Glycolysis, **(D)** glycolytic reserve, **(E)** ATP rate index and **(F)** ATP production rate was determined based on the ECAR profile under different conditions. BEAS-2B cells were exposed to DEP (100 or 300 μg/mL) or 0.5% DMSO (control) for 72 h. Data represent 3–5 independent experiments and are expressed as mean ± SD; *p < 0.05, ***p < 0.001, and ****p < 0.0001 significant difference between indicated groups.

We studied whether DEP exposure modifies mitochondrial respiration and glycolysis (Krabbendam et al., 2020). By measuring the oxygen consumption rate, we observed that exposure of BEAS-2B cells to 300 μg/mL DEP for 72 h reduced basal and ATP-linked respiration compared to control conditions (Figures 2A, B). Additionally, exposure to 100 or 300 μg/mL DEP for 72 h decreased maximal respiration and spare capacity compared to control (Figures 2A, B). Glycolysis is an important source of ATP in cells (Krabbendam et al., 2020) and was analyzed by measuring the extracellular acidification rate (ECAR). Our results reveal that exposure of BEAS-2B cells to 100 or 300 μg/mL DEP for 72 h significantly reduced the glycolytic capacity compared to that of control cells and that 300 μg/mL DEP significantly reduced the glycolytic reserve (Figures 2C, D). DEP exposure did not alter the ATP rate index (MitoATP/GlycoATP) nor ATP production (Figures 2E, F).

### DEP induces oxidative stress

We assessed the mRNA expression of the oxidative stress marker, thioredoxin, key players in the antioxidant response (Gpx-1, CAT, HO-1, HO-2, SOD-1 and SOD-2), and the transcription factor Nrf1 in BEAS-2B cells exposed to 100 μg/mL DEP or DMSO (control) for 24, 48 and 72 h. DEP exposure for 48 and 72 h did not significantly alter the mRNA expression of any of the genes studied (data not shown). DEP exposure for 24 h did not significantly increase Nrf1, however did increase thioredoxin mRNA levels (Table 1). Surprisingly, Gpx-1 mRNA expression was reduced in 24 h DEP treated cells while HIF-1α mRNA expression was increased (Table 1). As expected, HO-1 and HO-2 mRNA levels were significantly increased (Table 1). CAT and SOD-1 mRNA expression were not affected by DEP exposure for 24 h (Table 1). On the other hand, SOD-2 mRNA levels were significantly increased as compared to control (Table 1). Moreover, DEP exposure for 24 h did not change the protein level of HIF-1α (Supplementary Figure S2). SOD-1 is mainly found in the cytoplasm, whereas SOD-2 is exclusively expressed in mitochondria. These findings indicate that exposure to DEP might induce alterations in mitochondrial function.

**TABLE 1:** Exposure of BEAS-2B cells to DEP induced oxidative stress markers and differentially affected expression of key players of the antioxidant response system.

Genes	Control deltaCq (mean ± SD)	DEP deltaCq (mean ± SD)	DEP fold change (2^ΔΔCT^ ± SD)
Nrf1	18.28 ± 3.62	17.94 ± 3.94	1.28 ± 0.30
Thioredoxin	15.11 ± 4.38	14.33 ± 4.38	1.72 ± 0.26**
Gpx-1	13.29 ± 0.53	13.64 ± 0.59	0.78 ± 0.034***
HIF-1α	10.02 ± 1.12	9.78 ± 1.21	1.17 ± 0.07*
HO-1	20.80 ± 1.90	19.75 ± 2.20	2.11 ± 0.47*
HO-2	19.77 ± 2.31	18.41 ± 2.00	2.68 ± 0.88*
CAT	13.96 ± 0.86	14.14 ± 1.09	0.90 ± 0.25
SOD-1	18.04 ± 4.31	17.99 ± 4.38	1.05 ± 0.24
SOD-2	18.29 ± 5.20	17.73 ± 4.99	1.48 ± 0.26*

Gene expression levels of Nrf1, thioredoxin, Gpx-1, HIF-1α, HO-1, HO-2, CAT, SOD-1 and SOD-2 were analyzed by real-time quantitative PCR in BEAS-2B cells exposed to 100 μg/mL DEP or 5% DMSO (control) for 24 h. Data is expressed as fold over the control (vehicle treated) condition using the ΔΔCt method. Data represent 3 independent experiments and are expressed as mean ± SD; *p < 0.05 and ***p < 0.001, significant difference between indicated groups.

### DEP exposure does not affect BEAS-2B cell viability but induces inflammatory cytokines

**FIGURE 3:**
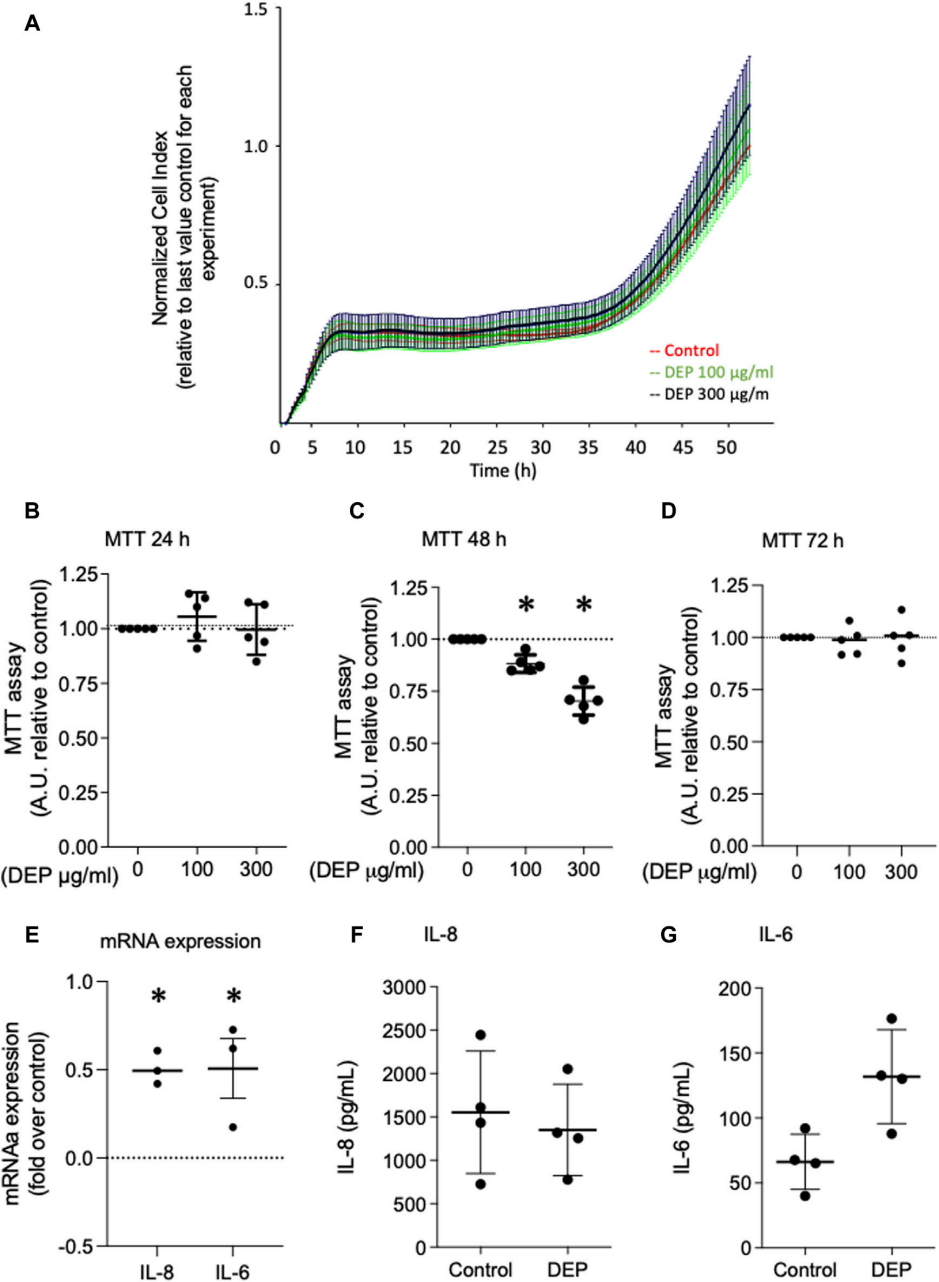
Exposure of BEAS-2B cells to DEP did not alter cell viability and induces expression of inflammation markers. **(A)** xCELLigence measurement in BEAS-2B cells exposed to DEP (100 or 300 μg/mL) or 5% DMSO (control) for 24 h. **(B–D)** MTT measurement of BEAS-2B cells exposed to the indicated concentrations of DEP for 24, 48 and 72 h. Exposure of BEAS-2B cells to DEP induces expression of inflammation markers. **(E)** IL-8 and IL-6 gene expression and **(F,G)** secreted protein was determined by real-time quantitative PCR and ELISA, respectively, in BEAS-2B cells exposed to 100 μg/mL DEP for 24 h. Real-time quantitative PCR data is expressed as fold over the control (vehicle treated) condition using the ΔΔCt method. Data are representative of 3–5 independent experiments and expressed as mean ± SD; *p < 0.05, significant difference between indicated groups.

We investigated the effects of 24 and 48 h DEP exposure on cell viability using MTT and xCELLigence (real-time) assays. Exposure of BEAS-2B cells to 100 μg/mL DEP for 24 h did not alter cell viability as measured by either assay (Figures 3A, B). However, the exposure of BEAS-2B cells to 100 or 300 μg/mL DEP reduced cell viability as measured by MTT after 48 h (Figure 3C). DEP exposure did not alter cell viability as measured by MTT after 72 h (Figure 3D). We also investigated the effect of DEP on the secretion of inflammatory cytokines. Exposure of BEAS-2B cells to 100 μg/mL DEP for 24 h increased the levels of IL-8 and IL-6 mRNA (Figure 3E) but did not alter IL-8 and IL-6 secreted protein compared to control (Figures 3F, G). DEP exposure for longer periods of time (up to 72 h) had no effect on IL-8 and IL-6 mRNA and protein (data not shown).

### DEP differentially alters the mRNA expression profile of downstream cAMP-effectors

**FIGURE 4:**
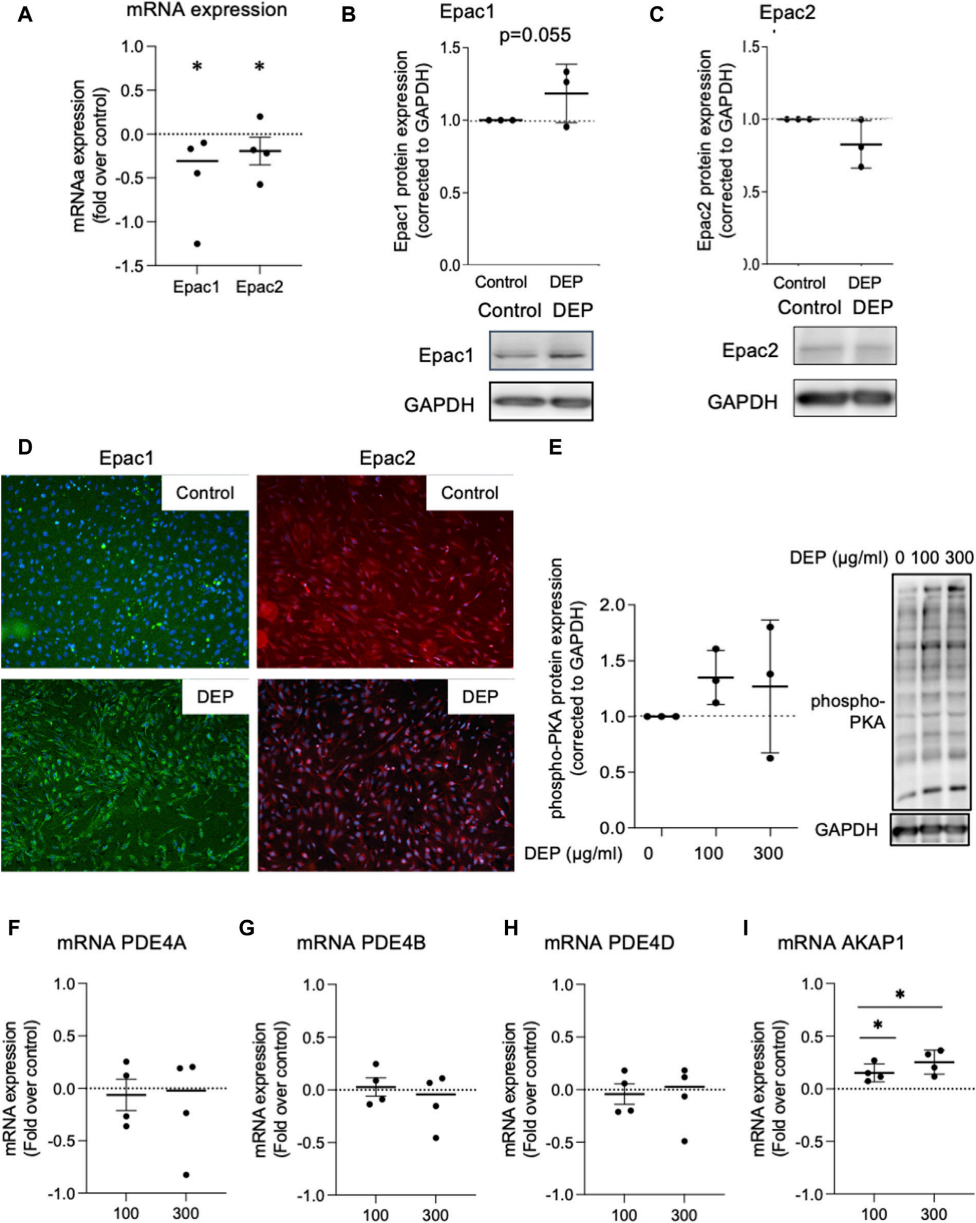
Exposure of BEAS-2B cells to DEP alters Epac mRNA content but does not affect expression of phospho-PKA substrates and PDE4 isoforms mRNA. **(A)** Gene expression levels of Epac1 and Epac2 were assessed using real-time quantitative PCR. Data is expressed as fold over the control (vehicle treated) condition using the ΔΔCt method. **(B,C,E)** Protein expression of Epac1, Epac2 and phospho-PKA substrates were analyzed using Western blotting. **(D)** Representative immunofluorescence images are shown. Scale bar of 200 μm. **(F–I)** Gene expression levels of PDE4A, PDE4B, PDE4D and AKAP1 were assessed using real-time quantitative PCR. BEAS-2B were exposed to 100 μg/mL DEP or 0.5% DMSO (control) **(A–D)**, unless indicated otherwise **(E–I)**, for 24 h. Data represent 3–4 independent experiments and are expressed as mean ± SD; *p < 0.05, significant difference between indicated groups.

We investigated the effects of DEP exposure on key elements of cAMP intracellular signaling since our group previously found that different stressors, including cigarette smoke, altered the expression of cAMP signaling proteins, including EP receptors, PDEs and Epac’s (Oldenburger et al., 2014a; Oldenburger et al., 2014b; Poppinga et al., 2015; Zuo et al., 2020a). We studied the effects of DEP (100 μg/mL, 24 h) on the expression of Epac1 and Epac2 in BEAS-2B cells. Exposure to DEP significantly reduced both Epac1 and Epac2 mRNA levels (Figure 4A). Other than a trend (*p* = 0.055) for a modest increase in Epac1 protein in whole cell lysates, little to no change was observed on Epac1 and Epac2 protein expression as assessed by Western blotting and immunofluorescence (Figures 4B–D). DEP exposure did not alter the basal levels of phospho-PKA substrates (Figure 4E) nor did it alter PDE4A, PDE4B or PDE4D mRNA expression (Figures 4F–H). The AKAP family member AKAP1 is a mitochondrial scaffold protein linked to cAMP compartmentalization and mitochondrial function (Hiura et al., 2000; Zhao et al., 2009; Merrill and Strack, 2014). Interestingly, our results show that exposure of BEAS-2B cells to DEP for 24 h concentration-dependently elevated AKAP1 mRNA (Figure 4I).

### DEP increases expression of β_2_-AR and EP4 mRNA but decreases cAMP signaling

**FIGURE 5:**
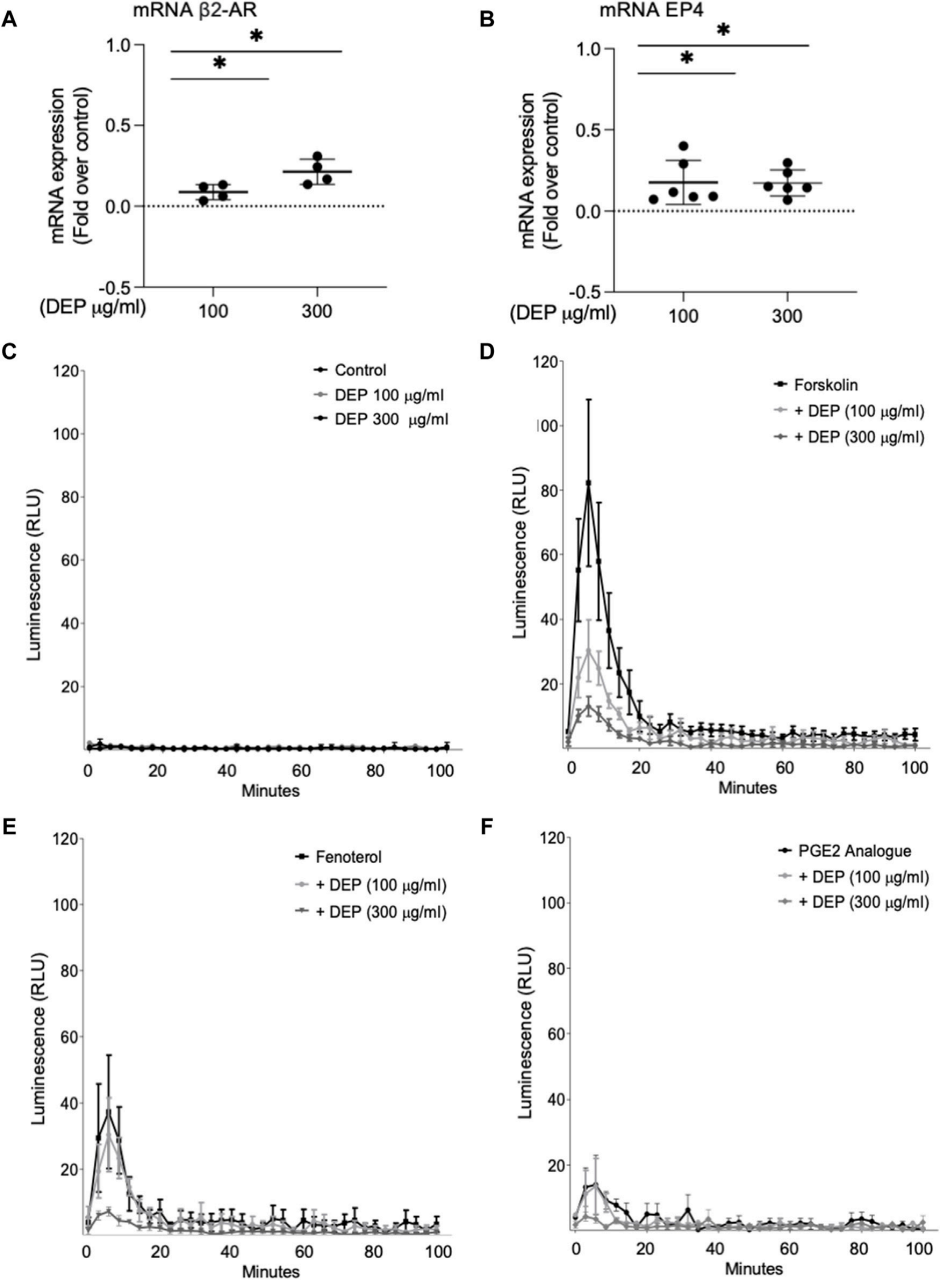
Exposure of BEAS-2B cells to DEP alters β_2_-AR, EP4 and cAMP kinetics. BEAS-2B cells were exposed to DEP (100 or 300 μg/mL) or 0.5% DMSO (control) for 24 h. **(A,B)** Gene expressions of β_2_-AR and EP4 were measured using real-time quantitative PCR. Data is expressed as fold over the control (vehicle treated) condition using the ΔΔCt method. **(C–F)** cAMP was measured in real-time using the GloSenor cAMP assay in the absence and presence of either forskolin, fenoterol (each 10 µM) or a PGE2 analogue (3 µM). The increase in luminescence signal was expressed as RLU; relative luminescence units. Data represent 2–6 independent experiments and are expressed as mean ± SD; *p < 0.05, significant difference between indicated groups.

We examined the impact of DEP exposure on the expression of two key GPCR coupled to cAMP signaling in BEAS-2B cells, β_2_-AR and EP4. Exposure to either 100 or 300 μg/mL DEP (24 h) increased β_2_-AR (Figure 5A) and EP4 (Figure 5B) mRNA levels. Expression of EP2 and EP3 mRNA were unchanged (data not shown). We then used the GloSensor cAMP assay to measure cAMP in real-time in BEAS-2B cells treated with agonists for these receptors. Exposure to 100 or 300 μg/mL DEP for 24 h did not alter basal cAMP levels (Figure 5C). Direct activation of ACs by forskolin resulted in increased luminescence that peaked at ≤10 min and rapidly declined to almost basal levels within 40 min in control cells (Figure 5D). Exposure of BEAS-2B cells to DEP (100 or 300 μg/mL) for 24 h reduced the forskolin-stimulated luminescence in a concentration-dependent fashion. Exposing cells to the β_2_-AR agonist, fenoterol, resulted in an increase in the luminescence signal peaking at ∼6 min and declining to basal levels within 40 min (Figure 5E). 24 h DEP exposure markedly inhibited fenoterol-stimulated cAMP levels, particularly at 300 μg/mL (Figure 5E). The EP receptor agonist, 16,16-dimethyl prostaglandin E2 (PGE2 analogue), stimulated an increase in luminescence that was approximately 50% of that induced by fenoterol but that also peaked at ∼6 min and declined to basal levels within 20 min (Figure 5F). Treatment with 300 μg/mL DEP diminished cAMP responses stimulated by the PGE2 analogue. Our data suggest that while DEP exposure increases mRNA expression of β_2_-AR and EP4, it actually impairs cAMP signaling by these GPCR by reducing the cellular capacity for cAMP generation.

### DEP decreases mRNA expression of AC9

**FIGURE 6:**
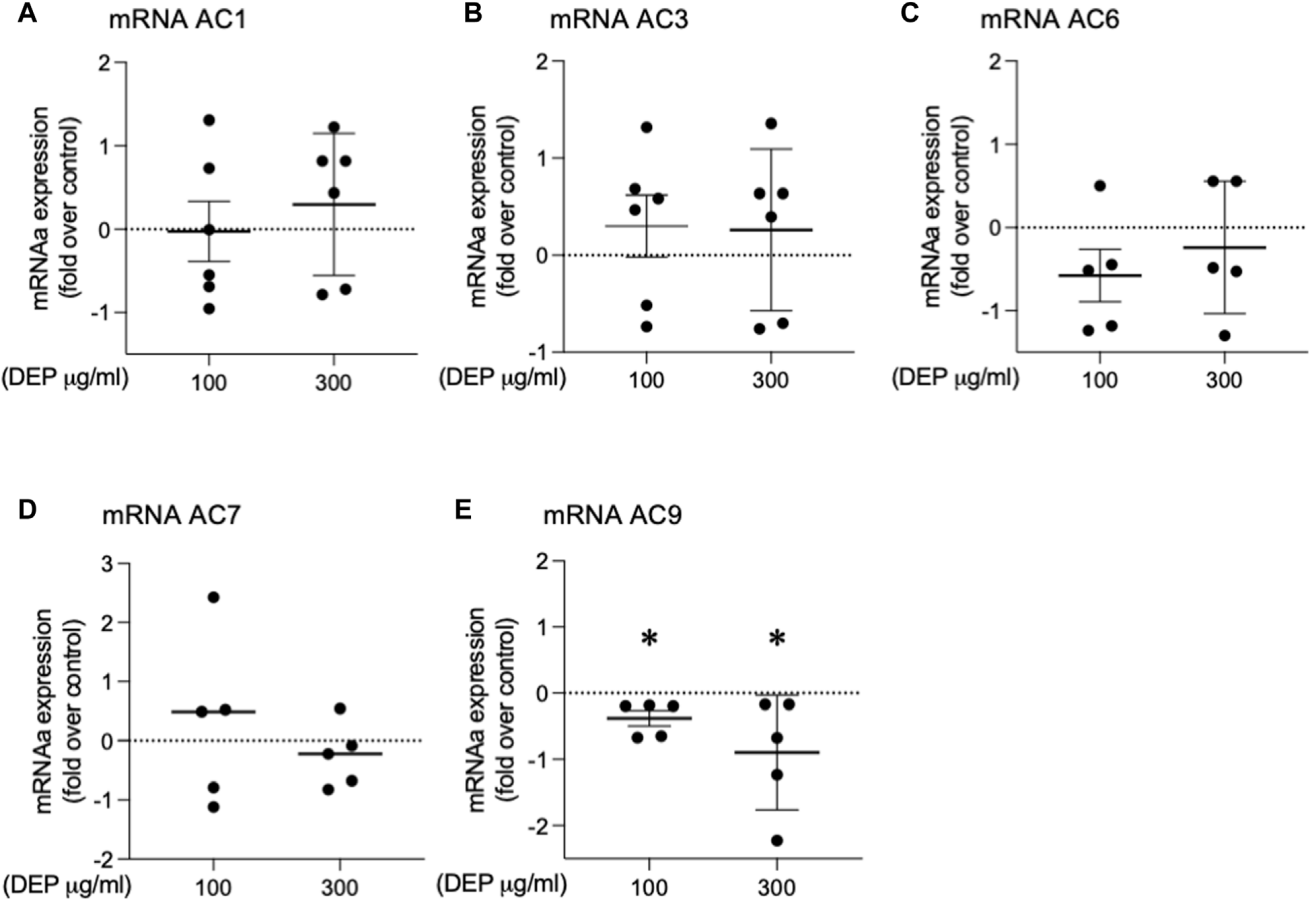
Exposure of BEAS-2B cells to DEP alters specific AC mRNA content. BEAS-2B cells were exposed to DEP (100 or 300 μg/mL) or 0.5% DMSO (control) for 24 h. **(A–E)** Gene expressions of AC1, AC3, AC6, AC7, AC9 were measured using real-time quantitative PCR. Data is expressed as fold over the control (vehicle treated) condition using the ΔΔCt method. Data represent 4–6 independent experiments and are expressed as mean ± SD; *p < 0.05, significant difference between indicated groups.

It is well established that whole lung tissue, including epithelial cells, express at least eight of the nine AC family members (Pierre et al., 2009; Halls and Cooper, 2017; Ostrom et al., 2022). We assessed the effect of DEP exposure on the expression of AC isoforms. 24 h exposure of BEAS-2B cells with 100 or 300 μg/mL DEP significantly decreased AC9 mRNA but had no effect on AC1, AC3, AC6 or AC7 mRNA levels (Figure 6). We were unable to detect mRNA for AC2, AC4, AC5 or AC8 in BEAS-2B cells (data not shown). Thus, DEP exposure reduces the expression of a specific AC isoform, AC9, possibly explaining the impaired cAMP signaling we observe.

## Discussion

We investigated potential alterations in mitochondrial function and the components of cAMP signaling in BEAS-2B cells exposed to DEP. We show that DEP exposure alters mitochondrial morphology, switching from category I (elongated mitochondria) to category II (fragmented mitochondria) with reductions in number of branches and branch length. These data are consistent with the idea that DEP exposure interferes with mitochondrial function. DEP exposure also increases the expression of genes associated with oxidative stress and inflammation. Furthermore, DEP exposure significantly reduces Epac1 and Epac2 mRNA content. Interestingly, we found a trend towards an increase in Epac1 protein content and hypothesize that a potential feedback mechanism between Epac1 protein and mRNA expression levels could occur. No significant effects on basal phospho-PKA substrates were detected. These observations suggest that DEP exposure differentially impacts the expression of downstream cAMP effectors.

We used two doses for DEP exposure, 100 and 300 μg/mL. An *in vivo* dosimetric assessment was conducted in a highly polluted area in Southern California to evaluate the deposition of total particulate matter (PM) and PM_2.5_ (fine particles with a diameter <2.5 μm) in an exposed adult. The study revealed that the concentration of DEP considered significant in this context ranged from 0.2 to 20 μg/cm^2^, corresponding to 1–100 μg/mL based on the authors’ calculations (Li et al., 2003). While 300 μg/mL is considered a high dose, it remains pertinent if one considers that diesel motors are less prevalent in the United States than other parts of the world. All urban areas are characterized by significantly increased concentrations of air pollution sources, often exceeding the air quality guideline levels established by the World Health Organization (WHO, 2006).

AKAP1 acts as a mitochondrial scaffold protein linking cAMP signaling and mitochondrial function (Hiura et al., 2000; Zhao et al., 2009; Merrill and Strack, 2014). DEP exposure induced AKAP1 (mRNA) in BEAS-2B cells, which was paralleled by decreased mitochondria network integrity evidenced by decreased branching and overall branch length. AKAP1 is localized in the outer membrane of mitochondria and plays a crucial role in preserving mitochondrial respiratory chain function while also regulating mitochondrial dynamics. Recent research highlights the multifaceted role of AKAP1: in a hyperoxia-induced acute lung injury model, AKAP1 deletion exacerbated inflammation by triggering autophagy and morphological changes in mitochondria (Narala et al., 2018). Conversely, disparate findings emerge from studies on human cancer cells and tissue samples, where AKAP1 is notably overexpressed, including in breast, prostate, and lung cancer tissues (Rinaldi et al., 2017). These contrasting outcomes underscore the context-dependent effects of AKAP1 across different disease models. We observe that the elevation of AKAP1 mRNA levels may be linked to the alteration in the mitochondria function of BEAS-2B cells exposed to DEP. Fragmentation of mitochondria is indicative of mitochondrial dysfunction and increased oxidative stress (Wu et al., 2011; Dolga et al., 2013). Mitochondrial branch length is a crucial parameter for assessing fusion and/or fission dynamics, as well as cellular adaptation to stress, offering valuable insights into mitochondrial health and cellular homeostasis (Mirzapoiazova et al., 2019; Kritskaya et al., 2024). Alterations in mitochondrial morphology frequently coincide with cellular dysfunction and disease. DEP resulted in a reduction in mitochondrial branch length, suggesting potential changes in mitochondrial dynamics, such as decreased fusion or increased fission activity, associated with modifications in cellular processes including energy production and apoptosis regulation.

Indeed, DEP exposure not only reduced basal and ATP-linked mitochondrial respiration but altered mitochondrial spare capacity as well. This is of particular interest since the mitochondrial spare capacity is a measure of the cellular ability to adapt to increased energy demands under stress conditions (Dolga et al., 2013). Previous reports demonstrate that exposure of mouse alveolar macrophages (Zhao et al., 2009) or RAW 264.7 macrophages (Hiura et al., 2000; Cattani-Cavalieri et al., 2020) to DEP and DEP extract from a light-duty diesel source, respectively, reduces mitochondrial membrane potential. Importantly, DEP exposure of BEAS-2B cells also reduced the ability to shift the energy production to glycolysis, leading to decreased basal glycolysis. *In vivo* exposure to air particulate matter for over 3 months induced a metabolic shift, decreasing glycolytic intermediates and enhancing *de novo* synthesis of fatty acids in mice (Reyes-Caballero et al., 2019). However, to the best of our knowledge, the present study is the first to detail the effects of DEP on specific mitochondrial functions, including the glycolytic reserve.

Oxidative stress and inflammation play a central role in air pollution-associated lung disorders (Xu et al., 2013; Park et al., 2016; Zhang et al., 2017; Kim et al., 2020). Exposure of BEAS-2B cells to DEP induced oxidative stress as indicated by induction of thioredoxin, HO-1 and SOD-2. It is important to note that SOD-2 serves as a mitochondrial matrix enzyme (Ighodaro and Akinloye, 2018) so an increase in SOD-2 points to an alteration in mitochondrial function. In agreement with our study, exposure of primary bronchial epithelial cells to aerosolized DEP for 24 h elevated HO-1 gene expression (Ji et al., 2018). Exposure to DEP also increased mRNA of the inflammatory cytokines IL-8 and IL-6. These findings are in line with previous observations that 24 h exposure of A549 cells to increasing concentrations of DEP upregulates gene expression of IL-6 and IL-8 (Vattanasit et al., 2014), and that DEP induced proinflammatory genes, including IL-6 and IL-8, in BEAS-2B (Bach et al., 2014). However, there was no increase in the protein levels of IL-8 and IL-6. Understanding why there is an elevation in mRNA expression without a concurrent increase in protein secretion is crucial to unraveling post-transcriptional regulatory mechanisms or potential cellular responses to DEP exposure. Additional studies will be necessary to elucidate the complex interplay between transcriptional and translational regulation in response to DEP exposure and its potential implications for lung-related conditions.

**FIGURE 7:**
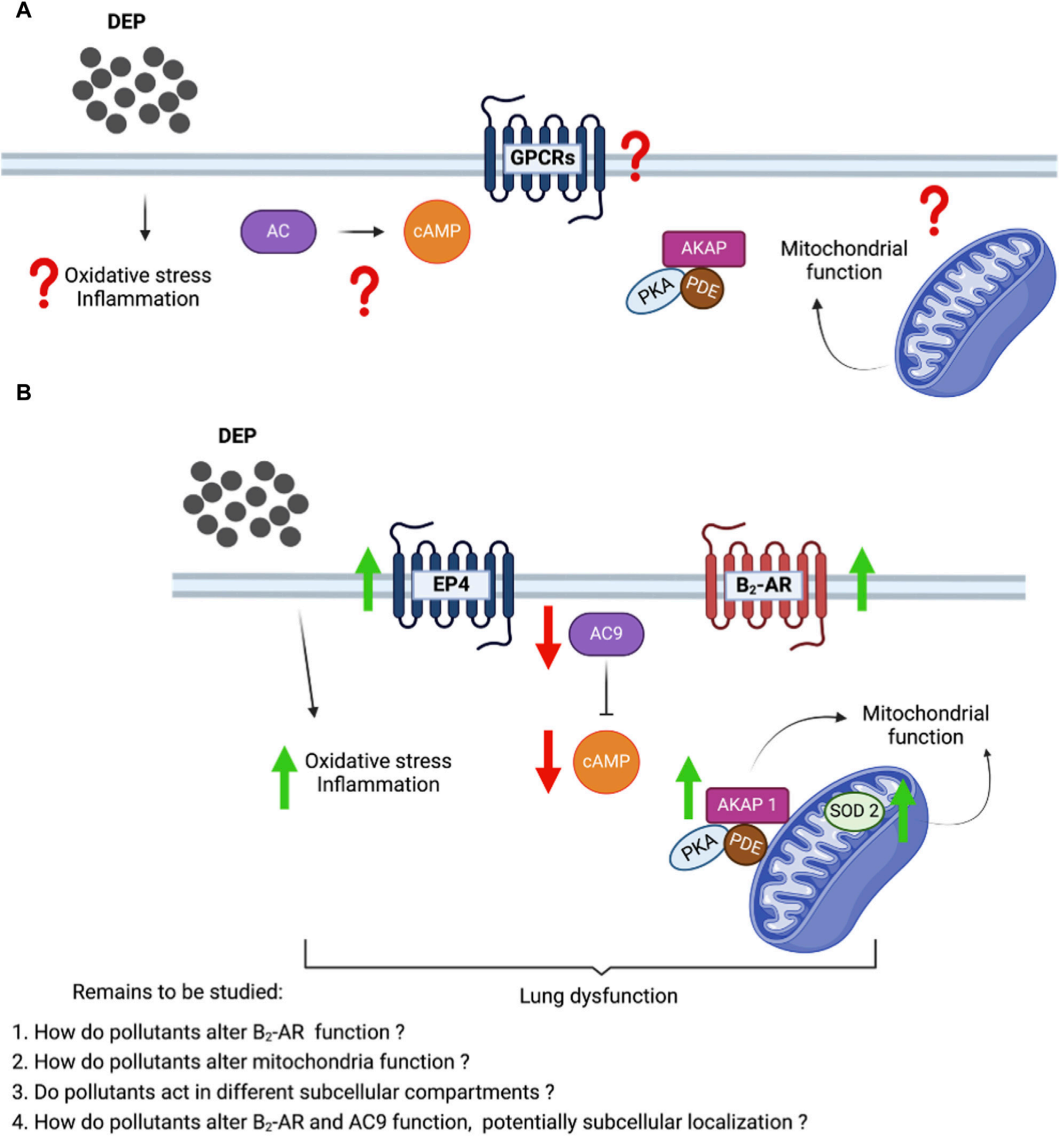
Proposed molecular mechanism showing the effect of DEP in the cAMP production and mitochondrial bioenergetics in human bronchial epithelial cells. **(A)** Unknown effects of DEP on inflammation and oxidative stress, GPCRs and cAMP signaling, and mitochondrial function. **(B)** DEP induces oxidative stress and inflammation; and induced cAMP generating receptor (β_2_-AR and EP4). However, the reduction of AC9 affected the cAMP production levels. AKAP1 and SOD2 elevation show the potential alteration in the mitochondrial function.

Mitochondria are the primary source of ROS but are also involved in several cell signaling events, participating in the mediation of inflammation in several inflammatory diseases (Lopez-Armada et al., 2013). The alteration of SOD-2 and the increase in inflammatory markers (IL-6 and IL-8) imply that mitochondria play a central role in the effects of DEP on BEAS-2B cells. Based on our current findings and the existing literature, it is highly likely that exposure to DEP induces both oxidative stress and an inflammatory response in BEAS-2B cells, which collectively contribute to lung dysfunction (Figure 7). It is important to highlight that cellular oxidative stress can trigger mitochondrial apoptotic pathways. DEP extract induces apoptosis in macrophages and generated ROS, which play a role in the apoptotic process (Hiura et al., 2000). Cadmium, a heavy metal pollutant, promoted an increase in intracellular ROS levels in BEAS-2B cells, thereby fostering oxidative stress and elevating the apoptotic rate, through the activation of the mitochondria-mediated intrinsic apoptosis pathway (Cao et al., 2021). While our studies do not focus on apoptosis, we have observed that exposure to DEP induces oxidative stress and alters mitochondrial function. Consequently, we suggest that DEP exposure indirectly impacts apoptosis in BEAS-2B cells.

DEP exposure affected cAMP signaling dynamics by impairing the cellular capacity to generate cAMP, which was paralleled by severe alterations in mitochondrial bioenergetics (Figure 7). These effects of DEP exposure could potentially contribute to lung dysfunction caused by air pollution. Recently, our group showed that cAMP compartments link β_2_-AR to members of the AKAP superfamily in air pollutant-exposed BEAS-2B cells (Zuo et al., 2020b). Exposure of BEAS-2B to DEP induces the oxidative stress marker SOD-2, known to tether to the mitochondrial matrix (Ighodaro and Akinloye, 2018). These specific observations provided us with the first indication that DEP bear the potential to alter mitochondrial function. The primary objective of this research was not to extensively explore the role of AKAP1, but to establish this mitochondrial scaffold protein as a potential link between mitochondrial cAMP compartmentalization and mitochondrial function (Merrill and Strack, 2014; Liu et al., 2020). Further investigation is needed to fully understand the link between the DEP-induced alterations in cAMP producing capacity and mitochondrial dysfunction.

cAMP signaling is coordinated by several members of the AKAP family and the regulation of cellular functions by cAMP seems to occur through activation of distinct intracellular effectors, including PKA and Epac1 and Epac2 (Schmidt et al., 2013; Poppinga et al., 2014). In an acute animal model of lung disease, Epac1 inhibits remodeling, whereas Epac2 promotes airway inflammation (Oldenburger et al., 2014a). We previously reported that expression of Epac1 (but not Epac2 and PKA) is sensitive to oxidative stress induced by air pollutants (Oldenburger et al., 2014a). Exposure of BEAS-2B cells to DEP increased several markers of oxidative stress (e.g., thioredoxin, HO-1, SOD-2), and decreased mRNA levels of Epac1 and Epac2. More studies are needed to unravel the potential mechanisms involved in the sensing of oxidative stress by Epac1. This ability appears to rely on the exogenous trigger (irritant) as it differs between exposure to DEP and other air pollutants.

PDEs are enzymes that hydrolyze cyclic nucleotide second messengers, promoting the compartmentalized regulation of cAMP (Zuo et al., 2019b). An important family of PDEs are the PDE4 isozymes, which are the targets of current therapy for respiratory disease such as COPD. In the present study, we show that exposing BEAS-2B cells to DEP did not alter the expression of PDE4 isoforms. Further investigations are needed to understand the role of other PDE isoforms in the DEP-induced alterations in cAMP.

The primary source of cAMP production induced by β_2_-AR occurs predominantly at the plasma membrane and AC9 seems to serve as significant mediator in β_2_-AR signaling. In HEK239 cells, AC9 traffics to endosomes when activated by β_2_-AR and contributes to cellular cAMP generation initiated by the activation of β_2_-AR in endosomes (Lazar et al., 2020). DEP exposure decreased AC9 mRNA levels but increased β_2_-AR and EP4 mRNA. DEP reduced cAMP levels stimulated by the AC activator, forskolin, the β_2_-AR agonist, fenoterol, or the stable EP4 agonist (Figure 7). It is well established that AC is the rate-limiting step in the cAMP signaling pathway (Ostrom et al., 2000). Our data support the notion that AC content is the primary driver of the magnitude of cAMP responses and suggest that the increase in receptor expression may be a “rescue” mechanism of the cell to try and compensate for the reduced capacity to produce cAMP. However, it is possible that other AC isoforms are involved in the reduced cAMP signaling we observe. AC9 is relatively insensitive to forskolin alone so a drop in AC9 expression would not be predicted to result in reduced responses to forskolin unless there was concomitant activation of Gs (Baldwin et al., 2019; Ostrom et al., 2022). DEP treatment led to high variability in several AC isoform mRNA levels (particularly AC7) so it is unclear if expression of other ACs were altered at the protein level.

Considering that cAMP is well-recognized to be beneficial in the regulation of lung function (Poppinga et al., 2014; Insel et al., 2019), an impaired capacity to produce cAMP could be highly detrimental to the maintenance of pulmonary function and contribute to features of lung dysfunction induced by air pollution. Indeed, it has been reported that cAMP generating receptor function is repressed in diseased lungs (Jones et al., 2019; Zuo et al., 2020a). Thus, it is tempting to speculate that the reduced ability to produce cAMP represents a mechanism by which DEP may cause damage to lung epithelial cells. Our study acknowledges certain limitations, notably the absence of longer time points for DEP exposure and a broader range of DEP concentrations. Addressing these limitations could provide a more comprehensive understanding of the impact of DEP on lung epithelial cells, encompassing mitochondrial function and cAMP signaling. Consequently, these areas deserve further investigation. Future studies are warranted to define the molecular nature of DEP-induced lung damage in systems more closely resembling intact lung physiology, such as polarized epithelia, organoids or precision cut lung slices from humans.

Oxidative stress and inflammation are pivotal players in the pathogenesis of various lung diseases, including asthma and COPD. Exposure to air pollution, such as DEP, triggered oxidative stress in BEAS-2B. This oxidative stress not only disrupts cellular homeostasis but also impairs mitochondrial function, compromising energy production and exacerbating cellular dysfunction (Cattani-Cavalieri et al., 2020). Moreover, alterations in cAMP signaling pathways may contribute to the pathophysiology of lung diseases. Dysregulation of cAMP levels can disrupt various cellular processes, including inflammation and immune cell function. The interplay between oxidative stress, inflammation, mitochondrial dysfunction, and cAMP modification underscores the complex nature of lung diseases. Understanding these interconnected pathways is essential for developing targeted therapeutic strategies aimed at reducing oxidative damage, suppressing inflammation, restoring mitochondrial function, and modulating cAMP signaling to alleviate the burden of lung diseases on affected individuals.

## Conclusion

We demonstrate that DEP exposure promotes oxidative stress and inflammation in BEAS-2B cells. Importantly, DEP leads to alterations in mitochondrial function and the capacity to generate cAMP. Our study is the first to provide detailed insights on the impact of DEP on cAMP signaling and mitochondrial function in human epithelial cells, facilitating the development of new therapeutic strategies to effectively target decline in lung function induced by exposure to air pollution.

## Data Availability

The original contributions presented in the study are included in the article/Supplementary Material, further inquiries can be directed to the corresponding author.
