# Supplementary material for: Diesel exhaust particles alter mitochondrial bioenergetics and cAMP producing capacity in human bronchial epithelial cells
Source: Front Toxicol. 2024 Jul 25;6:1412864. doi: 10.3389/ftox.2024.1412864 (PMC11306203; doi:10.3389/ftox.2024.1412864)
Supplement: Supplementary file 1 [file DataSheet1.pdf]

Supplemental table 1. Cq mean and standard deviation of control group.

	**Control Cq mean ± SD**	
IL-8	2.42 ± 1.32	n=3
IL-6	6.07 ± 1.15	n=3
Epac1	21.53 ± 1.32	n=3
Epac2	19.15 ± 2.05	n=3
PDE4A	13.38 ± 0.80	n=4
PDE4B	16.36 ± 0.49	n=4
PDE4D	18.77 ± 6.96	n=4
AC1	16,80 ± 6,45	n=6
AC2	ND	-
AC3	13,53 ± 6,16	n=6
AC4	ND	-
AC5	ND	-
AC6	17,55 ± 8,26	n=6
AC7	15.77 ± 1.751	n=5
AC8	ND	-
AC9	15.59 ± 7.191	n=4

Supplemental table 2. Primer sequences

Gene	Forward	Reverse	Accession number
**18s**	CGCCGCTAGAGGTGAAATTC	TTGGCAAATGCTTTCGCTC	NR_003286.4
**SDHA**	GGGAAGACTACAAGGTGCGG	CTCCAGTGCTCCTCAAAGGG	NP_004159.2
**RPL13A**	ACCGCCCTACGACAAGAAAA	GCTGTCACTGCCTGGTACTT	
**Catalase (CAT)**	CCATCGCAGTTCGGTTCT	GGGTCCCGAACTGTGTCA	NP_001743.1
**Glutathione peroxidase 1 (Gpx-1)**	CAACCAGTTTGGGCATCAG	GTTCACCTCGCACTTCTCG	NM_000581.4
**Heme oxygenase-1 (HO-1)**	GGGTGATAGAAGAGGCCAAGA	AGCTCCTGCAACTCCTCAAA	NP_002124.1
**Heme oxygenase-2 (HO-2)**	CTGAGGGAGTCGCTGACG	TTTCCACTTCCGCTGACAT	NP_002125.3
**HIF1-α**	ATCTCCATCTCCTACCCACA	CTTTTCCTGCTCTGTTTGGT	NP_001521.1
**Nrf1 (nuclear respiratory factor 1)**	CCATCTGGTGGCCTGAAG	GTAGTGCCTGGGTCCATGA	NP_005002.3
**SOD-1**	TCATCAATTTCGAGCAGAA	CAGGCCTTCAGTCAGTCCT	NP_000445.1
**SOD-2**	CTGGACAAACCTCAGCCCTA	TGATGGCTTCCAGCAACTC	NP_000627.2
**Thioredoxin**	TTACAGCCGCTCGTCAGA	AAGGCTTCCTGAAAGCAGTC	NP_003320.2
**Epac1 (RAPGEF3)**	GGAAGAACATGGCAAAGTGG	ATGAGCACTGGAATCTGGTC	NP_001092001.2
**Epac2 (RAPGEF4)**	AAGAACCATCAGGATGTCCG	TGTGGACTGGAGACAAACTG	NP_008954
**AKAP1**	CCAGTGCAGGAGGAAGAGTATG	CTCCCTCGACACCTCTATCCT	NP_003479.1
**EP2**	TGG CTA TCA TGA CCA TCA CC	TCC TTT CGG GAA GAG GTT TC	NP_000947.2
**EP3**	TAG CTC TTC GCA TAA CTG GG	GTT GCA GGA AAA GGT GAC TG	NP_942012.1
**EP4**	GAACATCCTGGCTTTTGAGC	TGTGACCACAATCCTCTGTC	NP_000949.1
**AC1**	CAT GAC CTG CGA CGA GGA CGA T	GTC CTG AAG CTG GTG GTA CTT T	NM_021116.4
**AC3**	CACGGGACCCAGCAAT	GCTCTAGGCCACCATAGGTA	NP_004027.2
**AC6**	GGCATTGATGATTCCAGCAAGAC	TGCAGGGCCTTAGGGACCAGA	NP_056085.1
**AC7**	TTAGCACATGATGAAAACAGACTT	CACTGGAGGGAAGAGATTTATG	NP_001105.1
**AC9**	CAAGTTCGACTCGGTGAACCT	CCGATGTAGAAGAGCGCATAC	NP_001107.2
**β_2-AR**	TTTTGGCAACTTCTGGTGCG	AGGCCTGACACAATCCACAC	NP_000015.2
**IL-6**	AAAGAGGCACTGGCAGAAAA	CAGGGGTGGTTATTGCATCT	NP_000591.1

**IL-8**	TAGCAAAATTGAGGCCAAGG	AAACCAAGGCACAGTGGAAC	NP_000575.1
**PDE4A**	GGGGTGAAGACCGATCAAGA	GGGGTGAAGACCGATCAAGA	NP_001104777.1
**PDE4B**	AATCTCACCAAGAAGCAGCG	AATCTCACCAAGAAGCAGCG	NM_002600.4
**PDE4D**	CACAGGTGGGCTTCATAGAC	AATCTCACCAAGAAGCAGCG	NP_001098101.1

Supplemental figure 1. Western blotting images of (A) Epac1, (B) Epac2 and (C) phosphor-PKA showing experiments 1 and 2, (D) phosphor-PKA showing experiment 3.

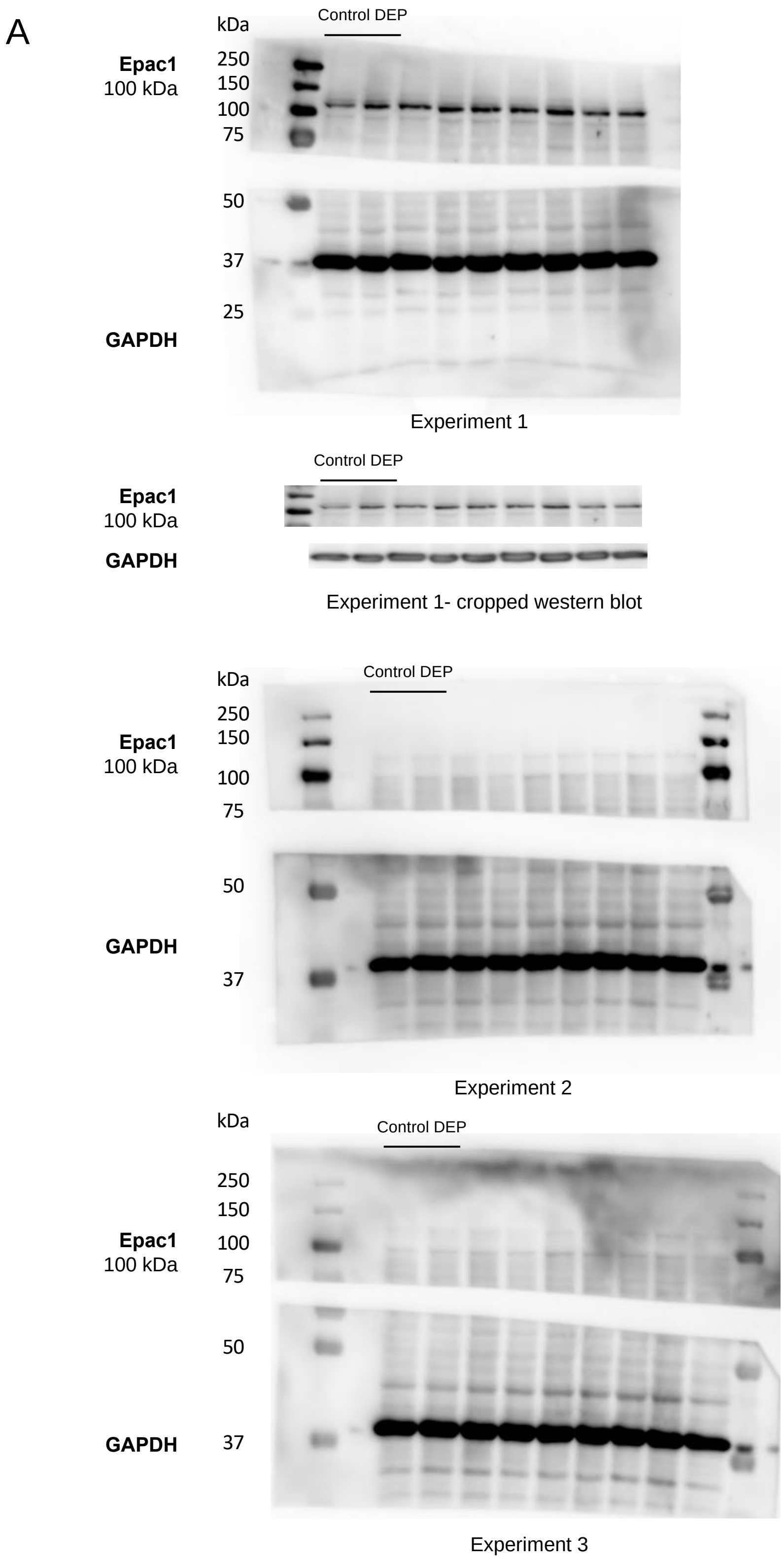

B

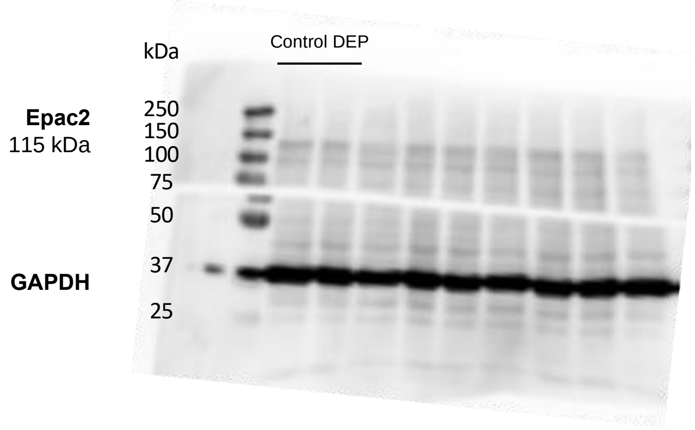

Experiment 1

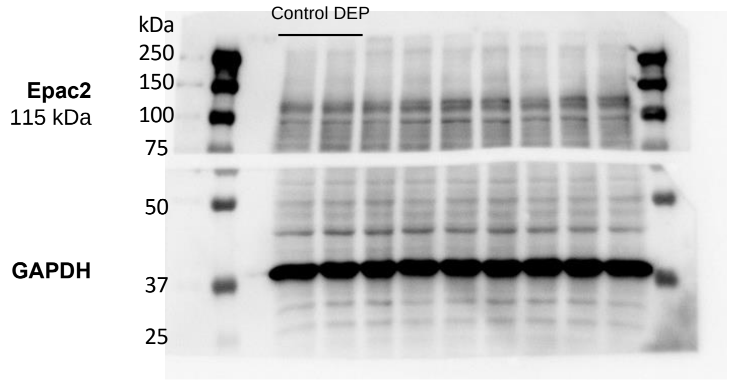

Experiment 2

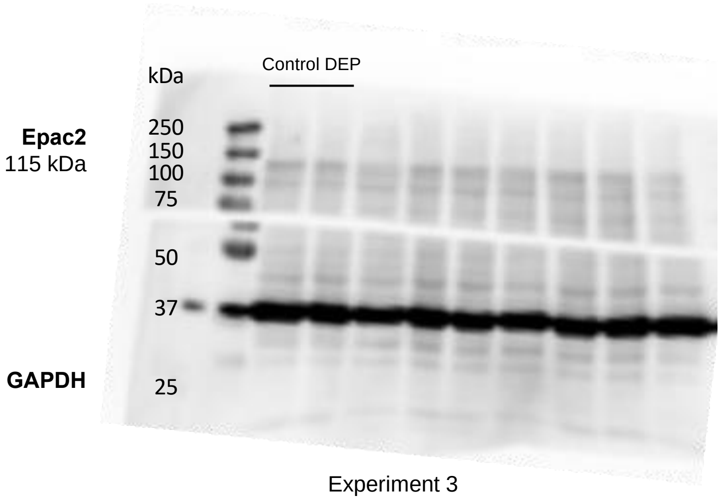

Experiment 3

C

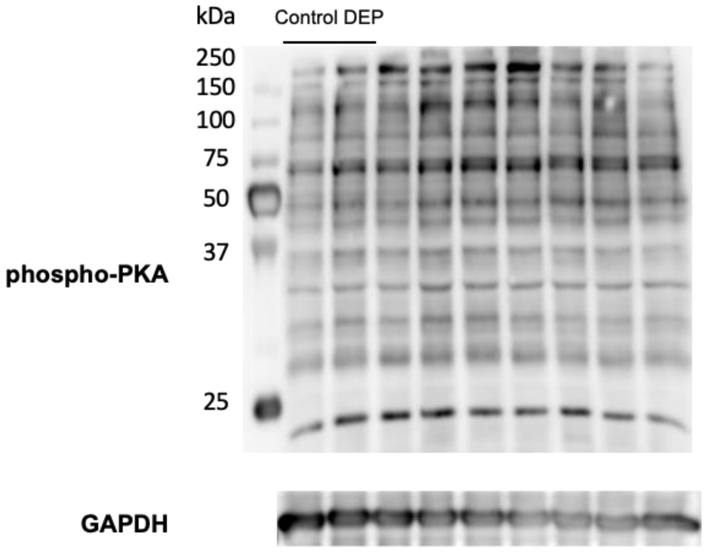

Experiment 1

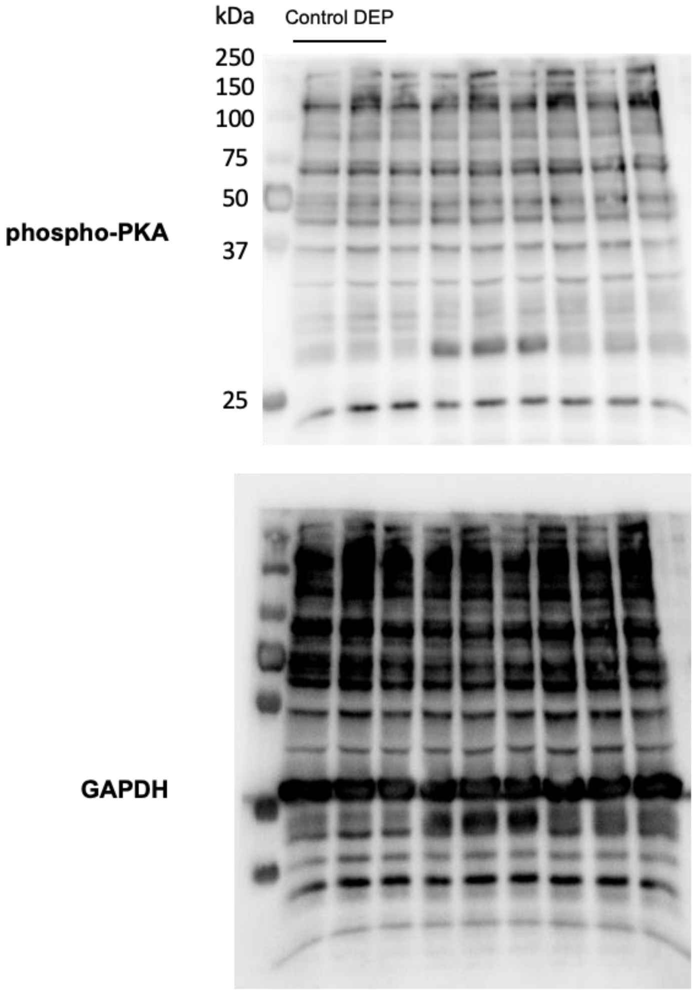

Experiment 2

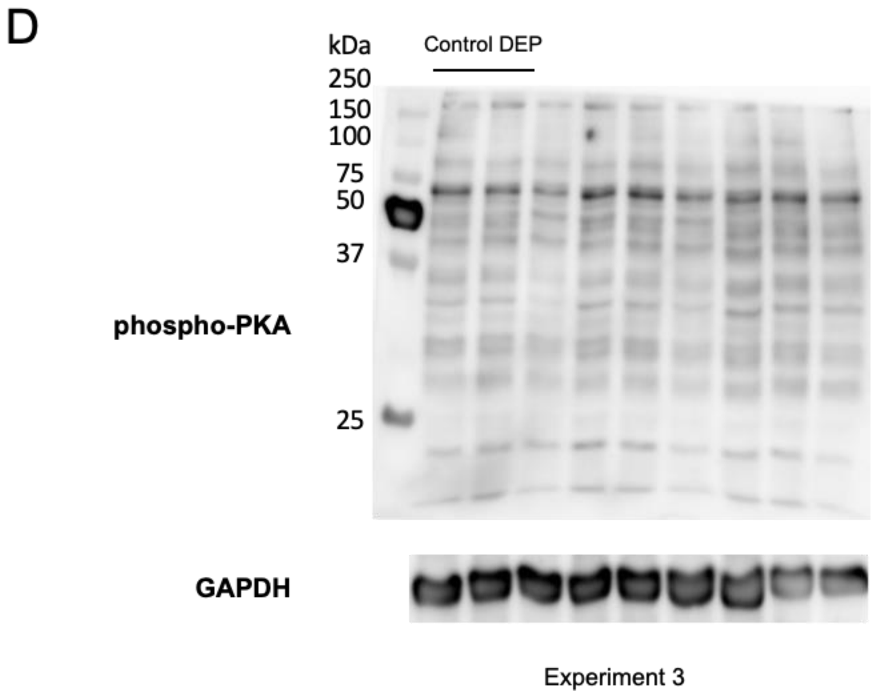

Supplementary figure 2. HIF-1α western blotting.

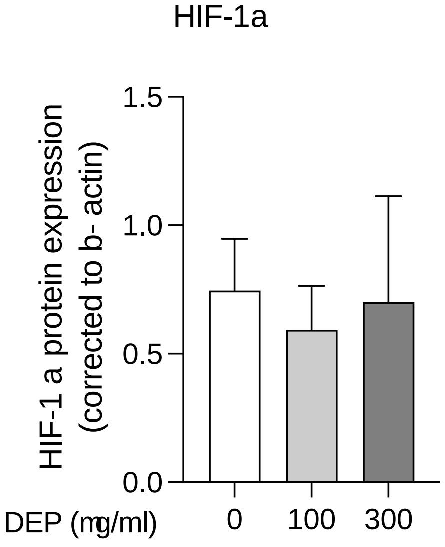

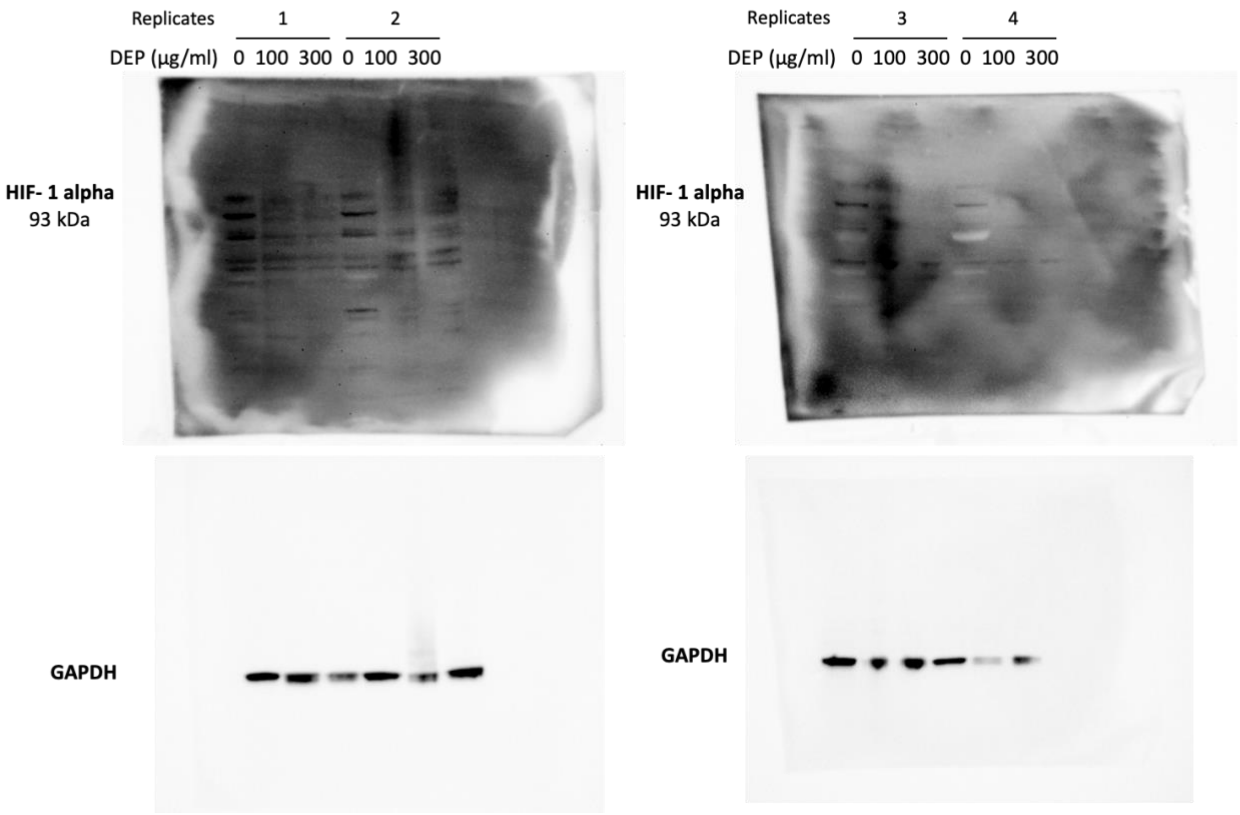

Supplementary figure 2. Exposure of BEAS-2B cells to DEP does not alter HIF-1α. Protein expression of HIF-1α was analyzed western blotting. BEAS-2B were exposed to 100 µg/ml DEP, 300 µg/ml DEP or 0.5% DMSO (control) for 24 h. Data represent 4 independent experiments and are expressed as mean ± SD; no significant difference between indicated groups.
